# Impact of Vanadium-Containing Stone Coal Smelting on Trace Metals in an Agricultural Soil–Vegetable System: Accumulation, Transfer, and Health Risks

**DOI:** 10.3390/ijerph20032425

**Published:** 2023-01-30

**Authors:** Zhichao Jiang, Xiyuan Xiao, Zhaohui Guo, Yunxia Zhang, Xiaoxiao Huang

**Affiliations:** School of Metallurgy and Environment, Central South University, Changsha 410083, China

**Keywords:** V-containing stone coal smelting, trace metal, soil–vegetable system, bioaccumulation factor, probability risk

## Abstract

Dietary exposure to trace metals (TMs) through vegetable consumption has been identified as a potential risk to human health. Fifty-one paired agricultural soil and leaf vegetable samples were collected around V-containing stone coal smelting sites in Hunan Province, China, to study the contamination and transfer characteristics of TMs (Cd, Cr, Cu, Pb, V, and Zn) in the soil–vegetable system. The health risk to local residents through vegetable ingestion was evaluated using Monte Carlo simulations. The results showed that 96.2%, 23.1%, 53.8%, 30.8%, 96.2%, and 69.2% of the soil samples had Cd, Cr, Cu, Pb, V, and Zn contents exceeding their related maximum allowable values, respectively. Cadmium and V were the primary pollutants based on the *I*_geo_ values. Moreover, 46.9% and 48.4% of vegetable samples exceeded the maximum permissible levels for Cd and Pb, respectively. There was a negative correlation between the bioaccumulation factors for Cd and V of the vegetable and soil physicochemical properties, including pH, organic matter, and free Fe_2_O_3_ content. Ingestion of garland chrysanthemum and pak choi posed high health risks, and Cd, V, and Pb were the primary contributors. These findings will help design strategies to minimize contamination and human exposure to soil–vegetable systems caused by V-containing stone coal smelting.

## 1. Introduction

Vanadium (V) is the fifth-most-abundant element in the Earth’s crust and has been widely used in modern industries, such as steel, catalysts, and pharmaceuticals [1]. Vanadium resources occur extensively in mineral and hydrocarbon deposits in Russia, South Africa, North America, and China [2]. In China, V ore resources, mainly stone coal and V-Ti magnetite, account for 43.2% of the world’s total reserves and 61.6% of global V production [3]. As associated minerals, stone coal is an essential V-containing resource [4], which accounts for >87% of the domestic V reserves located in China, and is widely distributed in the western region of Hunan Province [5]. V-containing stone coal has other metals, such as cadmium (Cd), chromium (Cr), and copper (Cu) [4]; thus, it could result in soil contamination from multiple trace metals (TMs) due to the smelting process. For example, the V, Cd, Cr, and lead (Pb) content in contaminated soil from stone coal smelting areas was 10.4, 5.64, 40.2, and 2.35 times higher than their corresponding local soil background values, respectively [6]. Soils contaminated with multiple TMs can degrade microbes, deteriorate the quality of surface water and groundwater, damage vegetable growth, and severely threaten human health [7,8]. Therefore, the potential hazards of V and other TMs in the soil around V-containing stone coal smelting sites require urgent attention. 

Vegetables grown in areas near a pollution source may readily accumulate high levels of TMs [9]. There are significant differences in the TM uptake and transfer capacities among different vegetable species [10,11]. Leafy vegetables often show a higher accumulation of TMs in their edible parts than rootstock, melon, and fruit vegetables due to the lack of barriers and the higher transportation of metals [12]. Chen et al. (2021) [10] indicated that despite the low content of Cd in soils, leafy vegetables could acquire higher Cd through their roots and translocate it to their aerial components, when grown in a greenhouse. Therefore, studies on TM contamination in leafy vegetables must be given priority attention. Moreover, the physicochemical properties of soil, such as pH, soil organic matter (SOM), and aluminum (Al)/iron (Fe) content, are crucial factors affecting the accumulation of TMs by vegetables [13]. It was reported that vegetables such as tomatoes and eggplants grown in greenhouse soils with a low pH, low organic matter content, and high phosphorus (P) content could accumulate a high amount of TMs [14,15]. Soil-to-vegetable transfer of TMs is a major pathway for human exposure to soil contamination. Many researchers reported that high health risks through vegetable ingestion can be found around industrial sites, such as non-ferrous smelting sites [16,17], mining areas [18], and E-waste sites [11]. The primary pollutants in soil–vegetable systems surrounding non-ferrous such as Cu and Pb/Zn smelting areas are As, Cd, Cu, Pb, and zinc (Zn) [7,17]. Notably, V, Cr, and Cu are considered as the major toxic pollutants caused by V-containing stone coal smelting [4,6]. This may lead to differences in the accumulation characteristics of TMs in surrounding soil–vegetable systems and critical factors affecting TM transfer as well as the ingestion risk. Nevertheless, more research is biased towards single V contamination, and no attention has been paid to other associated metal contamination in a V mining area and smelting area [8,19]. Thus, it is important to investigate the accumulation of V and other TMs in the soils and vegetables near stone coal smelting areas and assess human health risks via vegetable consumption. 

Previous investigations have focused on the ecological risk of vanadium ore pollution, agricultural soil polluted by TMs, and their potential health risks to local residents surrounding V-containing stone coal smelting areas [5,6,19]. However, information on the accumulation and transfer of V accompanied by other TMs in soil–vegetable systems as well as the vegetable ingestion risk caused by V-smelting activity has not been studied to date. Moreover, there is little information on the soil factors influencing the transfer of TMs in the soil–vegetable systems surrounding V smelting sites. Therefore, soil–vegetable samples were collected near V-containing stone coal smelters in order to: (1) evaluate the accumulation of Cd, Cr, Cu, Pb, V, and Zn in soils and leaf vegetables, (2) analyze the correlation between the physicochemical properties of soil and TM transfer in the soil–vegetable system, and (3) assess the probabilistic health risks of TMs via vegetable consumption. The results will provide a scientific basis for controlling the TM pollution of vegetables and soil remediation near V-containing stone coal smelters. 

## 2. Materials and Methods

### 2.1. Study Area and Sample Collection

**Figure 1:**
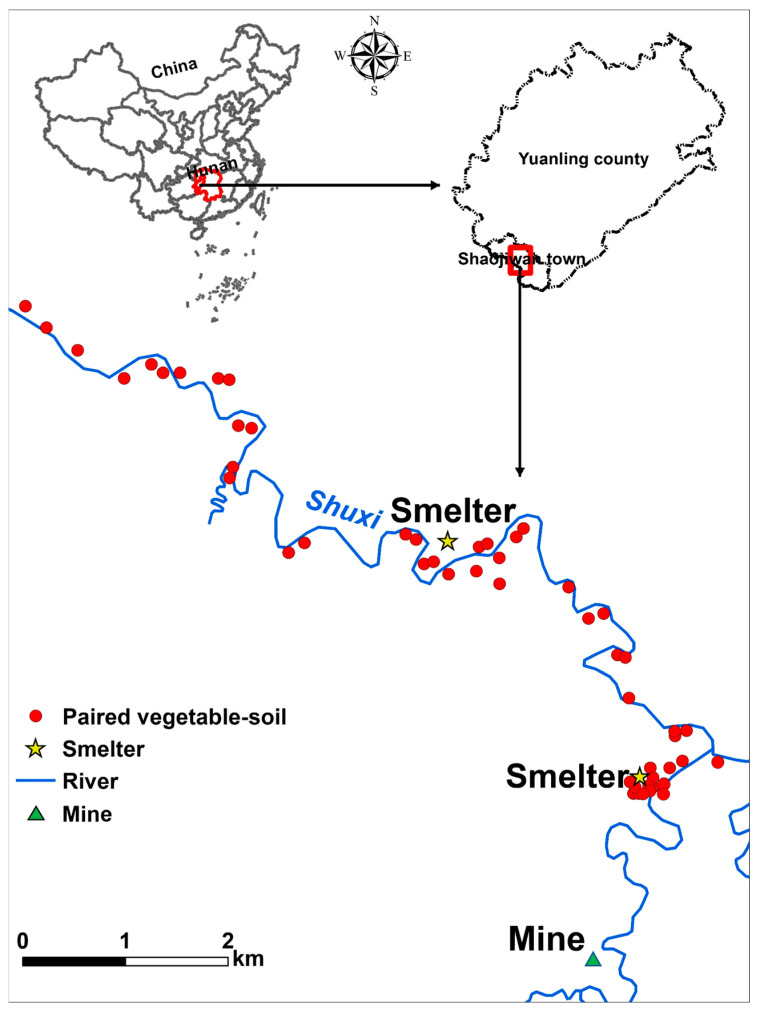
Study area and sampling sites.

The study area was located in Yuanlin County (latitude 28°12′33″, longitude 110°18′58″), western Hunan Province, China, in which two plants used for V extraction from stone coal were found (Figure 1). The sampling site has a mid-subtropical monsoon climate with a mean temperature and average annual precipitation of 16.7 °C and 1441 mm, respectively. The study area was far from highways with low traffic volume. The straight-line distance between the study area and two V-smelting sites was ~0.10–5.0 km. The two smelters were next to a river used for irrigation. Additionally, a small-scale Pb/Zn ore mine was located to the south of each of the two V-containing stone coal smelting sites by approximately 2 and 4.5 km, respectively, which may be another TM pollution source for the surrounding environment. A large area of farmland around the V smelters has been used to cultivate vegetables as a stable food source for local residents. 

Paired soil–vegetable samples (*n* = 51) were collected from the study area in April 2016. Five subsamples were collected at each site using a shovel and mixed into one composite soil or vegetable sample. Approximately 500 g of topsoil (0–20 cm depth) and 500 g of each species of leafy vegetable were collected at each sampling site. The leafy vegetables included asparagus lettuce (*Lactuca sativa* L.) (*n* = 11), pak choi (*Brassica chinensis* L.) (*n* = 6), coriander (*Coriandrum sativum* L.) (*n* = 5), flowering Chinese cabbage (*Brassica campestris* L.) (*n* = 11), garland chrysanthemum (*Chrysanthemum coronarium* L.) (*n* = 5), garlic sprout (*Allium sativum* L.) (*n* = 8), and lettuce (*Lactuca sativa* L.) (*n* = 5). These species of leafy vegetables are the main vegetables consumed by local residents. The edible part of the vegetable samples was collected, and any unhealthy outer leaves were excluded. The soil and vegetable samples were stored in polyethylene bags and were quickly transported to the laboratory for further analysis.

### 2.2. Sample Preparation and Analysis

Soil samples were air-dried at room temperature (25 °C), and any coarse materials and debris removed. The soils were sieved through a 10-mesh nylon sieve and ground to pass through a 100-mesh nylon sieve for metal content analysis. The vegetables were thoroughly washed using tap water, rinsed with deionized water, and dried with filter paper. The fresh and dry weights of the vegetable samples were recorded before and after being oven-dried at 60 °C to constant weight and were then ground into powder. 

The physicochemical properties of the soil samples were analyzed according to the general methods described by Lu [20]. The soil pH value was determined using a soil–water suspension (1:2.5, *w/v*) and pH meter (PHS-3D, Rex Instruments, Shanghai, China). The SOM content was determined using a volumetric method with K_2_Cr_2_O_7_. The free Fe_2_O_3_ (DCB-Fe) and free Al_2_O_3_ (DCB-Al) contents in soil samples were extracted using a dithionite citrate system buffered with sodium bicarbonate and assayed by atomic absorption spectroscopy (AAS, AAnalyst 800, PerkinElmer, Boston, MA, USA). The available potassium (K) content was extracted with 1.0 M NH_4_Ac and determined by flame photometry. The available P content was extracted with NaHCO_3_ solution and was determined using the molybdenum blue method [21]. The available nitrogen (N) content was measured using the alkaline hydrolysis method [5]. 

The vegetable and soil samples were digested with HNO_3_-HClO_4_ (9:1, *v/v*) and HNO_3_-H_2_O_2_ (2:1, *v/v*), respectively [17]. The metal contents were determined using inductively coupled plasma mass spectrometry (ICP-MS, Agilent 7500c, Agilent Scientific Technology Ltd., Santa Clara, CA, USA). To ensure the accuracy and precision of the determination, duplicate samples, reagent blanks, and standard reference materials (GBW-07406 for soils and GBW-10014 for vegetables) were analyzed with each digestion batch. The standard reference samples were obtained from the National Standard Research Center of China. The recovery rate of the TMs (Cd, Cr, Cu, Pb, V, and Zn) from the soil and vegetable samples was in the range of 90–110% of those of the standards, indicating the accuracy of our results. 

### 2.3. Environmental and Risk Assessment

#### 2.3.1. Soil Trace Metal Accumulation Assessment

The geo-accumulation index (*I*_geo_) was used to assess the accumulation level of TMs in soil [22], which was calculated using Equation (1):

(1) $$I_{\mathrm{geo}}=\log_{2}\left(\frac{C_{\mathrm{soil}}}{1.5B_{\mathrm{soil}}}\right)$$

where *C*_soil_ is the TM content (mg/kg) in the soil samples from the study area, and *B*_soil_ is the geochemical background value (mg/kg) of a given metal in soil (as shown in Table 1 as the BV). The constant 1.5 is a widely used coefficient representing the natural fluctuations of the soil background values [23]. The classes of *I*_geo_ values for identifying pollution levels of TMs in soils are listed in Table S1.

#### 2.3.2. Bioaccumulation Factor

The bioaccumulation factor (BAF) was used to evaluate the translocation ability of the TMs from soil to the edible parts of the vegetables [24], which was calculated as follows:

(2) $$\mathrm{BAF}=\frac{C_{\mathrm{vge}\left(fw\right)}}{C_{\mathrm{soil}}}$$

where *C*_veg(*fw*)_ is the TM content in vegetables (mg/kg, fresh weight), and *C*_soil_ represents TM content in soil (mg/kg). 

#### 2.3.3. Health Risk Assessment

Due to the lack of carcinogenic slope factors reported for TMs, the non-carcinogenic risk was estimated by calculating the hazard quotient (HQ). The hazard index (HI), which is the sum of HQs for different TMs, was used to evaluate the overall non-carcinogenic risk via vegetable ingestion. The HI values of <1 indicate the vegetables are safe to ingest. The HQ and HI were calculated using the following formulae [25,26]:

(3) $$\mathrm{CDI}=\frac{{\mathrm{IR}}_{\mathrm{vge}}\times C_{\mathrm{vge}\left(fw\right)}\times \mathrm{EF}\times \mathrm{ED}}{\mathrm{BW}\times \mathrm{AT}}$$

(4) $$\mathrm{HQ}=\frac{\mathrm{CDI}}{RfD}$$

(5) $$\mathrm{HI}=\sum {\mathrm{HQ}}_{i}$$

where CDI is the chronic daily intake of TMs (mg/kg per day); IR_vge_ is the daily ingestion of vegetables (g/day); *C*_veg(*fw*)_ is the TM content in the vegetables (mg/kg, fresh weight); EF is the exposure frequency (days/year), ED is the exposure duration (years); BW is the body weight (kg); AT is the average exposure time for non-carcinogenic effects (ED × 365 days/year); and R*f*D is the reference dose of a specific metal (mg/kg per day). The parameters required by the equations are listed in Tables S2 and S3.

### 2.4. Monte Carlo Simulation

The Monte Carlo simulation is commonly used to quantify the variability and uncertainty of risk assessments [27,28]. The simulation used the mean, standard deviation, and minimum and maximum values of the TM contents in vegetables. The data distribution was set to a log-normal distribution, and simulation was performed over 10,000 iterations. The simulated data on the TM contents were used to assess the health risk. 

### 2.5. Statistical Analysis

The data and figures were analyzed using OriginPro 2021 software (OriginLab, Northampton, ME, USA). The map of the sampling sites was drawn using ArcGIS 10.2 (ESRI, Redlands, CA, USA). Statistical analysis was performed using Excel 2019 (Microsoft, Redmond, WA, USA) and SPSS Statistics 25.0 software (IBM, Chicago, IL, USA). Principal component analysis (PCA) was used to reduce the number of factors that can show the origin of the TMs in the soil samples. One-way analysis of variance was adopted to study the difference in the *I*_geo_ values of TMs in the soil samples, BAF of TMs in vegetables, and health risk. Correlation analysis was conducted using the Pearson correlation. The significance level was set at *p* < 0.05. The probability distribution of HI was calculated using Crystal Ball 11.1 software (Oracle Corporation, Austin, TX, USA). The Kolmogorov–Smirnov test was used to check the normality.

## 3. Results and Discussion

### 3.1. Soil Physicochemical Properties 

The soil pH values were acidic to slightly alkaline, in the range of 4.57–7.22 with a geomean value of 5.79 (Table 1), which was close to the soil background pH value of 5.53 in Hunan Province [29]. The geomean contents of the DCB-Al and DCB-Fe in the soil samples were 0.66 and 1.55 g/kg, respectively. The SOM content ranged from 12.3 to 35.8 g/kg, with a geomean of 26.7 g/kg, which was slightly lower than the average SOM content (29.4 mg/kg) of cultivated soil in Hunan Province [30]. The content of available P in soil was low and in the range of 0.01–0.13 mg/kg, while a wide range of available content for N (28.0–90.8 mg/kg) and K (23.0–211 mg/kg) was observed. The geomean contents of available N, P, and K in soils were 56.9, 0.03, and 65.5 mg/kg, respectively, suggesting that the agricultural soil was suitable for vegetable growth. 

### 3.2. Evaluation of the Soil TM Content and Contamination

There was a great variation in the TM contents in the agricultural soil samples (Table 1). The Cd, Cr, Cu, Pb, V, and Zn contents in the soil samples were in a wide range, with 0.25–69.4, 61.6–728, 0.99–354, 31.0–446, 101–2434, and 119–1742 mg/kg, respectively. The geomean contents of Cd, Cr, Cu, Pb, V, and Zn in soil were 11.3, 121, 27.8, 88.2, 409, and 329 mg/kg, respectively. Furthermore, 96.2%, 23.1%, 53.8%, 30.8%, and 69.2% of the soil samples had Cd, Cr, Cu, Pb, and Zn contents higher than those recommended for TMs by the risk control standard for soil contamination of agricultural land (GB15618–2018) [31], respectively, and 96.2% of the soil samples exhibited V content higher than the guideline value of Canadian soil quality [32]. The variation coefficients of these TMs in the soil samples were >1, suggesting their intensive interference with human activities [33].

**Figure 2:**
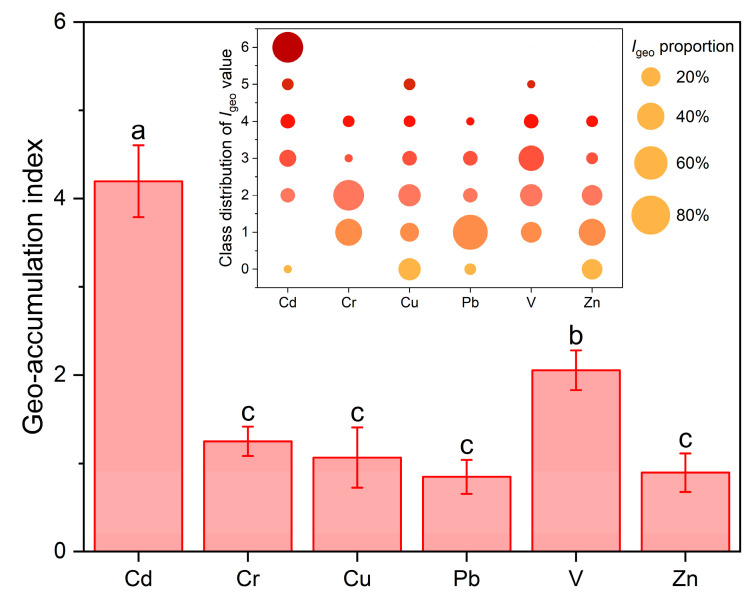
The *I*_geo_ values and proportions of trace metals in agricultural soils around the V-containing stone coal smelting site. Values followed by different lowercase letters are significantly different at α = 0.05.

The average *I*_geo_ values followed the order of Cd (4.20) > V (2.06) > Cr (1.25) > Cu (1.07) > Zn (0.89) > Pb (0.85), and those of Cd and V were significantly higher than those of the other metals (*p* <0.05) (Figure 2). The percentage of *I*_geo_ values observed for Cd above 1 accounted for 96.2% of the soil samples, indicating that most were moderately to extremely contaminated by Cd. The *I*_geo_ values of V, Cr, and Cu in 76.9%, 61.5%, and 53.8% of the soil samples exceeded the moderately contaminated level, respectively (Figure 2). Meanwhile, the percentage of *I*_geo_ values higher than 1 for Pb and Zn in the soil samples were only 26.9% and 38.5%, respectively, suggesting that the soil samples were slightly contaminated by these two metals. These results were in accordance with previous studies that reported Cd, V, and Cr or Cu were severely accumulated in soils surrounding V-containing stone coal smelting sites [6,34]. In general, Cd and V are the primary pollutants in the agricultural soils surrounding V-containing stone coal smelters. 

**Figure 3:**
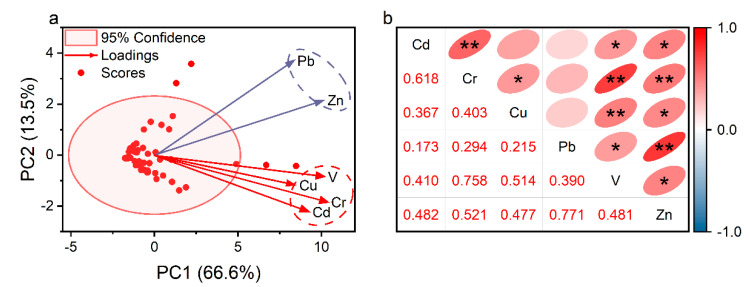
Principal component analysis (**a**) and correlation coefficient matrix (**b**) of trace metals in agricultural soils around the V-containing stone coal smelting site. *, *p* < 0.05. **, *p* < 0.01.

The PCA results showed that two principal components accounted for 80.1% of the total variance, with 66.6% and 13.5% explained by PC1 and PC2, respectively (Figure 3a). PC1 mainly represented Cd (38.5%), Cr (44.8%), Cu (36.1%), and V (44.0%). Furthermore, there was a significantly positive correlation among the Cd, Cr, Cu, and V contents in soil (*p* < 0.05) (Figure 3b). Cadmium, Cr, and Cu are the most commonly associated TMs in V-containing stone coal smelters [6], which may be volatilized into waste gas at a high temperature or coexist with V in wastewater and finally enter the surrounding soil [35]. A previous study also reported that the strong positive correlations among V, Cr, and Cu contents in soil were related to roasted stone coal slag [34]. PC2 represented Pb (74.8%) and Zn (44.6%) in their variances. The Pb content was significantly positively correlated with Zn content (*p* < 0.01), indicating they were derived from similar origins. The Pb/Zn ore mining activity nearby may be the essential pollution source of the soil Pb and Zn contamination in the soils surrounding these two V-containing stone coal smelters (Figure 1). It was reported that mining activities release considerably lower amounts of TMs than smelting [7]. This may explain the low accumulation of Pb and Zn in the soil samples. 

### 3.3. Trace Metal Content in Vegetables and Bioaccumulation Factor

**Figure 4:**
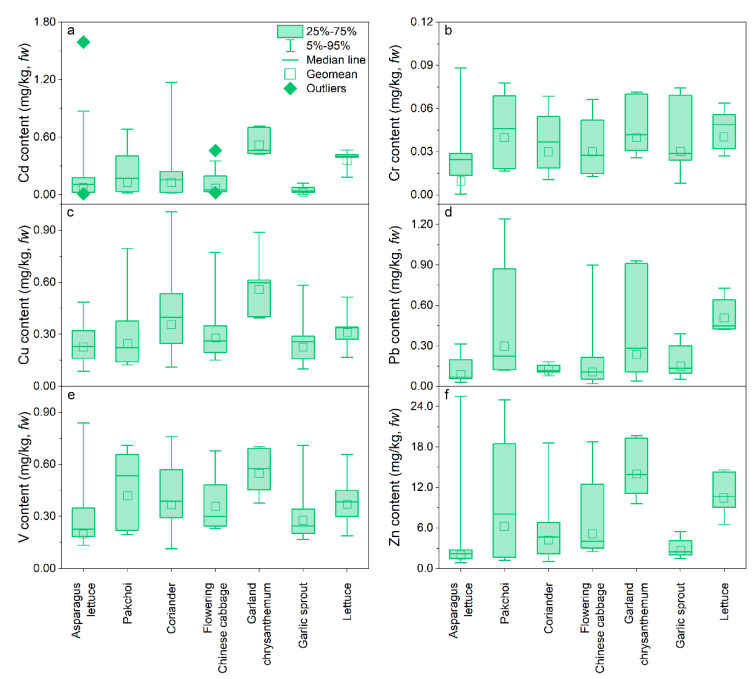
The trace metal contents in vegetables around the V-containing stone coal smelting site. (**a**–**f**) represents the content of Cd, Cr, Cu, Pb, V, and Zn, respectively.

Trace content in vegetables varies widely depending on the vegetable types and TMs. The Cd, Cr, Cu, Pb, V, and Zn contents in vegetables were in the range of 0.003–1.59, 0.001–0.09, 0.08–1.01, 0.001–0.09, 0.02–1.24, 0.07–0.84, and 0.79–25.5 mg/kg, respectively, with huge variations observed among the different vegetable species (Figure 4). The highest geomean Cd, Cu, V, and Zn contents of 0.52, 0.56, 0.55, and 14.0 mg/kg were found in garland chrysanthemum, and those of Cr and Pb were 0.04 and 0.51 mg/kg for lettuce, respectively. In contrast, garlic sprout had the lowest geomean contents of Cd (0.03 mg/kg) and Cu (0.23 mg/kg), and the lowest contents of Cr, Pb, V, and Zn were found in asparagus lettuce (0.01, 0.09, 0.20, and 2.09 mg/kg, respectively). This can be attributed to the different accumulation abilities of vegetables for TMs and varying soil conditions [36]. The high TM contents observed in garland chrysanthemums and lettuce were probably due to the high transpiration rate inherent in the large mass of their leaves, which could promote water and metal uptake by mass flow [37]. About 46.9% and 48.4% of the vegetable samples exceed the maximum permissible levels (MPLs) for Cd (0.20 mg/kg) and Pb (0.30 mg/kg) (GB2762–2017) [38], respectively. Garland chrysanthemum had a higher Cd content (*p* < 0.05), suggesting that it may threaten human health. The Cr content observed in the vegetables was below the MPLs (1.0 mg/kg). 

**Figure 5:**
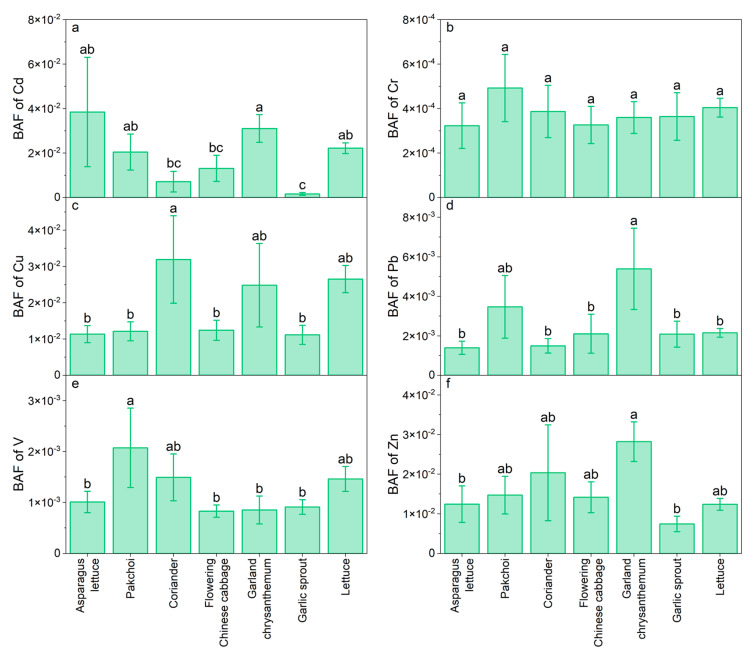
The bioaccumulation factor (BAF) of trace metals in vegetables around the V-containing stone coal smelting site. (**a**–**f**) represents the BAF of Cd, Cr, Cu, Pb, V, and Zn, respectively. Values followed by different lowercase letters are significantly different at α = 0.05.

The BAF values varied greatly among the vegetable species and TMs. The average BAF values of Cd (0.019), Cu (0.016), and Zn (0.014) in vegetables were higher and had similar values, while those of Pb (0.002), V (0.001), and Cr (0.0003) were very low (Figure S1). These results suggest that the studied vegetables preferred to accumulate Cd, which may endanger human health. Liu et al. (2021) [11] also observed that Cd had the highest BAF values among the TMs studied, implying that most of the Cd in soils could be taken up by various vegetables. Cadmium is a readily mobile metal, and its exchangeable content in soils is usually higher than that of other metals [39]. Copper and Zn are essential micronutrients for plant growth and can easily transfer from soil to the aboveground parts of vegetables via their root uptake [36]. Coriander and pak choi showed the highest BAF values for Cu and V, respectively. The BAF values of Cd, Pb, and Zn for garland chrysanthemum were higher than those of flowering Chinese cabbage and garlic sprout (Figure 5), which might be related to metal accumulation in the vegetables via atmospheric deposition [37,40,41]. In contrast, there was no significant variation in the BAF values for Cr among the different vegetables (*p* > 0.05). Similar previous results reported that the transfer factors of TMs in leafy vegetables were as follows: Cd > Zn > Cu > Cr > Pb; this indicated that there was a strong mobility of Cd from soil to leafy vegetables [10]. Therefore, better effective practices are required to reduce the accumulation of TMs with a high BAF. 

### 3.4. Influencing Factors for TM Transfer in Soil–Vegetable Systems

**Figure 6:**
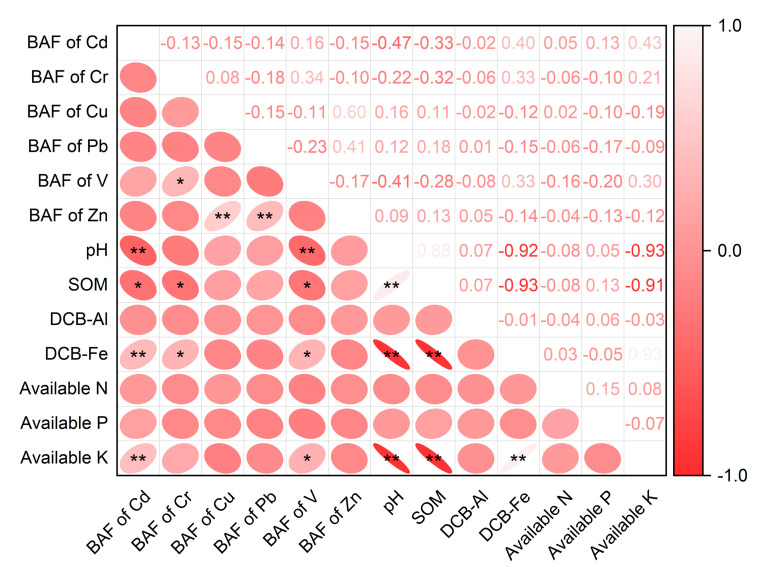
The correlation coefficient matrix of bioaccumulation factor (BAF) of trace metals and soil properties. *, *p* < 0.05. **, *p* < 0.01.

The soil properties, such as pH, SOM, and Al/Fe contents, greatly affect TM chemical speciation and bioavailability [42], thereby influencing the metal transfer from soil to vegetables. The BAF values of Cd, Cr, and V were positively correlated with the soil’s available K content (*p* < 0.05) (Figure 6). Potassium can enter plants via passive transport systems, and the existence of K^+^ channels can promote the migration of Cd, Cr, and V from the soil to crops in a synergistic manner [41,43,44]. In contrast, there was a significantly negative correlation between the BAF values of Cd and V and the soil physicochemical properties, including pH, SOM, and DCB-Fe (*p* < 0.05) (Figure 6). A decrease in soil pH value means more H^+^ is released into the soil solution, which can improve the availability of TMs [13]. This was in agreement with previous studies, which showed that soil pH could negatively affect the BAF of Cd in pak choi and the higher bioavailability and mobility of metals with increasing acidification [12]. SOM can act as a chelating agent or provide sorption sites for TMs, thus reducing their extractable content and immobilizing metals to the solid substances in soil [45,46]. DCB-Fe in soils can retain Cd and V via mineral adsorption, and Fe^2+^ may compete with Cd^2+^ for membrane transporters in the vegetable rhizosphere [13,47]. These results indicated that the soil properties greatly influenced the transport of Cd, Cr, and V in the soil–vegetable system, which was in agreement with previous studies [14,41]. However, the BAF values of Cu, Pb, and Zn showed no significant correlation with the soil properties, which may be due to the heterogeneity of soil texture and the sources of these metals [48]. Therefore, applying organic fertilizers or Fe-containing amendments to polluted agricultural soil may reduce the accumulation of Cd and V in vegetables [11].

There was a significant relationship between the total contents of Cd, Cr, Cu, and V in soils and the BAF values of the corresponding TMs in vegetables (*p* < 0.05), and the total Pb and Zn contents were not significantly correlated with their BAF values (Table S4). Similar previous results reported that the total contents of Cd and Cr in soils significantly correlated with those in flowering Chinese cabbage [49]. Soils polluted by industrial activity usually have high proportions of bioavailable contents of TMs [50,51], which lead to the total Cd, Cr, and Cu contents in soils being significantly related to the uptake ability of TMs in vegetables [49,52]. Our previous study also investigated that the average V content in the bioavailable fraction accounted for 25.5% of the total V in the soils surrounding V-containing stone coal smelting sites [53]. This indicated that a good correlation might be observed between soil total V and its BAF in vegetables. In contrast, the Pb and Zn content accumulated in soils from mining activities were mainly associated with the residual fraction [54], resulting in no significant relationship between these two metals and their BAF in vegetables. It is noteworthy that the bioavailable TM content in soils is also an important factor affecting TM uptake by leafy vegetables and usually shows a better correlation with the TM content in vegetables [14,42]. For example, Li et al. (2021) [49] found that CaCl_2_-extractable Cd, Ni, and Zn in soils better correlated with their contents in flowering Chinese cabbage, and Cd in various leafy vegetables was better related to DTPA-extractable Cd in soils [52]. These results suggested that the bioavailable TM content in soils would provide a better indication of the transfer ability of TMs from soil to vegetables, which should be investigated in our future studies.

The vegetable species is another significant factor contributing to the vegetable uptake of TMs besides soil properties [48,52]. As shown in Figure 5, coriander, pak choi, and garland chrysanthemum showed higher BAF values of Cd, Cu, Pb, V, and Zn than other leafy vegetables. Similar results were observed in previous studies, which showed that the BAF values of Cd, Cu, Pb, and Zn in coriander, pak choi, and garland chrysanthemum were relatively high [17,36,42]. This could be attributed to the different genotypes, rhizospheric microbial compositions, root-absorbing capacities, and electron translocation types for TMs in various vegetable species [41,42,55]. 

### 3.5. Non-Carcinogenic Risk Assessment through Vegetable Ingestion

**Figure 7:**
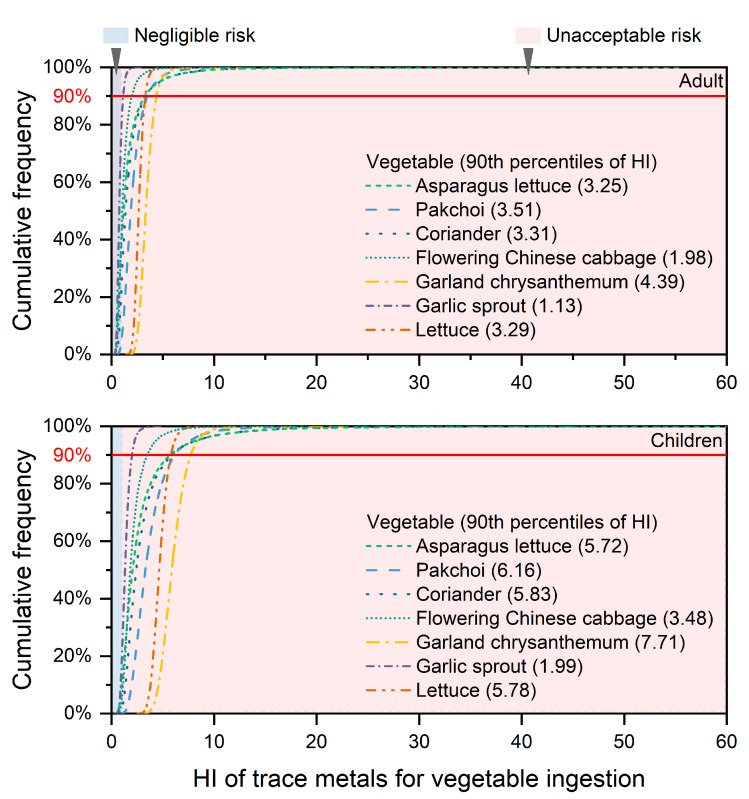
Probability distribution of hazard index (HI) caused by ingestion of vegetables grown in agricultural soils around the V-containing stone coal smelting site for adults and children.

We assessed the 90th percentiles of the HI that provides conservative and protective estimates to avoid underestimating the risk [56]. The HI varied markedly depending on the vegetable species, and the 90th percentile of the HI of the selected vegetables exceeded 1, indicating the ingestion risk from vegetable consumption (Figure 7). The 90th percentiles of the HIs for vegetables in children followed the order of garland chrysanthemum (7.71) > pak choi (6.16) > coriander (5.83) ≈ lettuce (5.78) ≈ asparagus lettuce (5.72) > flowering Chinese cabbage (3.48) > garlic sprout (1.99), which were significantly higher than those for adults (*p* < 0.05). This result implied that vegetables grown in contaminated soils pose a considerable health risk, and children are the vulnerable group. This was in agreement with the findings that leafy vegetables could accumulate high levels of TMs and thus pose a high health risk [57]. In this study, garland chrysanthemum, pak choi, and coriander showed relatively higher BAF values for most TMs and more severe health risks. Therefore, it is necessary to avoid consumption of the above-mentioned vegetables cultivated surrounding the V-containing stone coal-contaminated area. In addition, TMs in soil particles can enter the human body via other pathways, such as ingestion, dermal contact, and inhalation, when people are working in agricultural fields. Although these three pathways contribute less to the health risk than vegetable ingestion [56], their long-term impact on human health cannot be ignored. 

**Figure 8:**
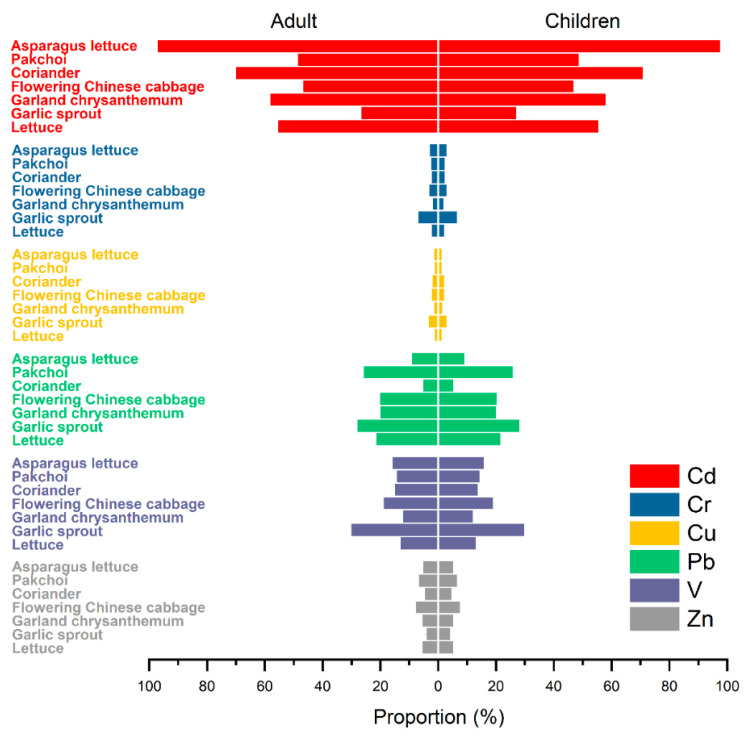
Risk contribution of trace metals to hazard index (HI) of vegetable ingestion for adults and children.

Among the studied TMs, Cd contributed the most to the non-carcinogenic risk, accounting for 26.8–70.2% and 27.2–71.1% in adults and children, respectively (Figure 8). Except for garlic sprout, the 90th percentiles of the HQ values of Cd for adults and children through the ingestion of selected vegetables were >1, indicating that Cd was the most important contaminant in leafy vegetables causing health risks (Table 2). These results may be due to the high Cd accumulation in soils and vegetables and high toxicity of Cd for humans. The contribution to the non-carcinogenic risk from Pb was in the range of 5.32–28.4%. The HQ values for Pb in pak choi and garland chrysanthemum were >1, indicating that humans were vulnerable to Pb exposure in these vegetables. Although the contribution from V to the HQ values ranged from 12.3% to 30.3% for the six vegetables (Figure 8), the HQ values of pak choi and garland chrysanthemum were >1 for children only, indicating that children were exposed to higher potential risks by ingesting vegetables contaminated with Cd, V, and Pb. This may be ascribed to children’s higher metabolism and absorption capacity when compared with adults [58]. In contrast, the contributions to the non-carcinogenic risk were lower for Cr, Cu, and Zn, which could be considered safe for people consuming these vegetables. The results were in agreement with previous studies, which showed that Cd and Pb were the major risk elements, followed by Cr, Cu, or Zn for leafy vegetables surrounding non-ferrous smelters [17,59]. In conclusion, the higher health risks of Cd/V-induced disease through vegetable ingestion may be caused by V-containing stone coal smelters and warrant more attention from researchers. 

## 4. Conclusions

This study investigated the contamination characteristics of TMs in soil–vegetable systems located around V-containing stone coal smelting areas and evaluated the health risks from vegetable ingestion. The agricultural soils were contaminated with multiple TMs. Specifically, 96.2%, 23.1%, 53.8%, 30.8%, and 69.2% of the soil samples had Cd, Cr, Cu, Pb, and Zn contents higher than the recommended values for TMs per the risk control standard for soil contamination of agricultural land (GB15618–2018), respectively. The V content in 96.2% of soil samples was higher than the guideline value of Canadian soil quality. Cadmium and V were the primary pollutants based on the *I*_geo_ values. About 46.9% and 48.4% of the vegetable samples exceeded the maximum permissible levels for Cd and Pb in vegetables (GB2762–2017), respectively. Garland chrysanthemum, pak choi, and coriander had higher BAF values for Cd, Cu, Pb, V, and Zn. Soil pH, SOM, and the DCB-Fe content were negatively correlated with the BAF values of Cd and V, which were also significantly affected by vegetable species and their total contents in soils. The 90th percentile of the HI values of the selected vegetables exceeded 1, and the primary contributors to non-carcinogenic risk were Cd (26.8–71.1%), V (12.3–30.3%), and Pb (5.32–28.4%). Garland chrysanthemum and pak choi posed potentially high risks to residents. Therefore, increased attention should be paid to the potential risks to human health through vegetable ingestion near V-containing stone coal smelting areas. 

## Figures and Tables

**Table 1:** The physicochemical properties and trace metal contents in agricultural soils around the V-containing stone coal smelting site (*n* = 51).

Parameter	Minimum	Median	Maximum	Arithmetic Mean (SD)	Geometric Mean (SD)	BV	SV	CV
Physiochemical properties								
pH ^a^	4.57	5.78	7.22	5.84 (0.76)	5.79 (1.14)	5.53	5.5–6.5	0.13
SOM (g/kg)	12.3	27.6	35.8	27.1 (4.24)	26.7 (1.21)	12.3	-	0.16
DCB-Al (g/kg)	146	0.63	2.84	0.80 (0.46)	0.66 (3.81)	0.44	-	0.58
DCB-Fe (g/kg)	537	1.69	5.46	1.87 (0.96)	1.55 (3.35)	0.67	-	0.52
Available N (mg/kg)	28.0	59.6	90.8	59.4 (17.1)	56.9 (0.36)	58.3	-	0.29
Available P (mg/kg)	0.01	0.03	0.13	0.04 (0.03)	0.03 (0.02)	0.03	-	0.77
Available K (mg/kg)	23.0	58.8	211	80.3 (52.6)	65.5 (1.92)	69.1	-	0.65
Trace metals								
Cd (mg/kg)	0.25	19.0	69.4	22.1 (19.6)	11.3 (5.31)	0.41	0.30	1.94
Cr (mg/kg)	61.6	106	728	151 (144)	121 (1.79)	33.9	150	1.19
Cu (mg/kg)	0.99	27.9	354	52.3 (75.9)	27.8 (3.64)	8.56	50.0	3.02
Pb (mg/kg)	31.0	64.8	446	115 (105)	88.2 (2.60)	32.7	90.0	1.12
V (mg/kg)	101	462	2434	563 (527)	409 (2.21)	95.6	130 ^b^	1.29
Zn (mg/kg)	119	266	1742	453 (434)	329 (2.17)	118	200	1.32

SD, standard deviation; BV, background values for agricultural soils in the sampling area; SV, *risk control standard for soil contamination of agricultural land in soil environmental quality of China* (GB 15618–2018). ^a^, pH value is dimensionless. ^b^, *Canadian soil quality guidelines for the protection of environmental and human health: summary tables*. -, not mentioned.

**Table 2:** Non-carcinogenic risk through vegetable ingestion around the V-containing stone coal smelting site for adults and children: 90th percentile of hazard quotient (HQ).

Vegetable Species	Adults						Children					
	Cd	Cr	Cu	Pb	V	Zn	Cd	Cr	Cu	Pb	V	Zn
Asparagus lettuce	2.73	0.09	0.04	0.26	0.45	0.15	4.81	0.15	0.07	0.45	0.79	0.27
Pak choi	2.08	0.11	0.06	1.11	0.62	0.29	3.66	0.19	0.11	1.95	1.09	0.50
Coriander	2.64	0.09	0.08	0.20	0.57	0.18	4.64	0.16	0.15	0.36	0.91	0.31
Flowering Chinese cabbage	1.13	0.08	0.06	0.49	0.46	0.19	1.98	0.13	0.10	0.86	0.81	0.33
Garland chrysanthemum	2.93	0.10	0.08	1.02	0.62	0.28	5.15	0.18	0.15	1.80	1.09	0.49
Garlic sprout	0.38	0.10	0.05	0.40	0.43	0.06	0.68	0.17	0.08	0.71	0.75	0.11
Lettuce	2.06	0.09	0.05	0.80	0.49	0.21	3.62	0.15	0.09	1.41	0.87	0.36

## Data Availability

All data and materials are available upon request.

## Supplementary Materials

The following supporting information can be downloaded at: https://www.mdpi.com/article/10.3390/ijerph20032425/s1. Figure S1: The bioaccumulation factor (BAF) of trace metals in vegetables around the V-containing stone coal smelting site. Values followed by different lowercase letters are significantly different at α = 0.05; Table S1: Seven classes of geo-accumulation index; Table S2: Description and values of factors used in risk assessment [11,60]; Table S3: Reference dose (R*f*D) of trace metals [60]. Table S4: Correlation coefficient matrix of the total content of trace metals (TMs) in agricultural soils and their BAF values for vegetables.
