# Supplementary material for: Impact of Vanadium-Containing Stone Coal Smelting on Trace Metals in an Agricultural Soil–Vegetable System: Accumulation, Transfer, and Health Risks
Source: Int J Environ Res Public Health. 2023 Jan 30;20(3):2425. doi: 10.3390/ijerph20032425 (PMC9915546; doi:10.3390/ijerph20032425)
Supplement: Supplementary file 1 [file ijerph-20-02425-s001.zip › ijerph-2175428-supplementary.pdf]

Supporting information

Title: Impact of vanadium-containing stone coal smelting on trace metals in an agricultural soil-vegetable system: Accumulation, transfer, and health risks

Author: Zhichao Jiang, Xiyuan Xiao *, Zhaohui Guo, Yunxia Zhang, Xiaoxiao Huang

Address: School of Metallurgy and Environment, Central South University, Changsha 410083, China

*Corresponding author

E-mail address: xiaoxy@csu.edu.cn

Pages = 7

Figure = 1

Tables = 4

List

1. **Figure S1** The bioaccumulation factor (BAF) of trace metals in vegetables around the V-containing stone coal smelting site. Values followed by different lowercase letters are significantly different at $\alpha = 0.05$.

2. **Table S1** Seven classes of geo-accumulation index.

3. **Table S2** Description and values of factors used in risk assessment **(Liu et al., 2021; USEPA, 2015)**.

4. **Table S3** Reference dose (R*f*D) of trace metals **(USEPA, 2015)**.

5. **Table S4** Correlation coefficient matrix of the total content of trace metals (TMs) in agricultural soils and their BAF values for vegetables.

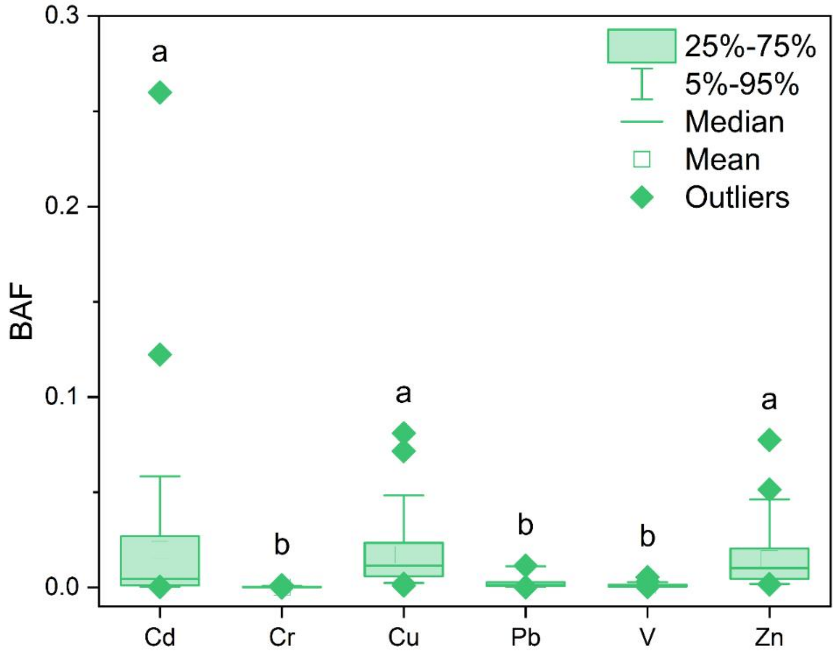

Figure S1 The bioaccumulation factor (BAF) of trace metals in vegetables around the V-containing stone coal smelting site. Values followed by different lowercase letters are significantly different at $\alpha = 0.05$.

Table S1 Seven classes of geo-accumulation index.

Class	Value	Soil quality
0	$I_{geo} \leq 0$	Practically uncontaminated
1	$0 < I_{geo} < 1$	Uncontaminated to moderately contaminated
2	$1 < I_{geo} < 2$	Moderately contaminated
3	$2 < I_{geo} < 3$	Moderately to heavily contaminated
4	$3 < I_{geo} < 4$	Heavily contaminated
5	$4 < I_{geo} < 5$	Heavily to extremely contaminated
6	$5 < I_{geo}$	Extremely contaminated

Table S2 Description and values of factors used in risk assessment **(Liu et al., 2021; USEPA, 2015)**.

Parameter	Abbreviation	Value	Unit
Body weight [a]	BW_a	60.0	kg
Body weight [c]	BW_c	26.0	kg
Vegetable ingestion rate [a]	IR_{vge}^{a}	244	g/day
Vegetable ingestion rate [c]	IR_{vge}^{c}	186	g/day
Exposure frequency	EF	365	day/year
Exposure duration [a]	ED_a	30	year
Exposure duration [c]	ED_c	7	year

[a] for adults; [c] for children.

Table S3 Reference dose (R*f*D) of trace metals **(USEPA, 2015)**.

Metal	R*f*D for ingestion (mg/kg-day)
Cd	1.00E-03
Cr	3.00E-03
Cu	4.00E-02
Pb	3.50E-03
V	5.00E-03
Zn	3.00E-01

Table S4 Correlation coefficient matrix of the total content of trace metals (TMs) in agricultural soils and their BAF values for vegetables.

BAF	Total TM contents
Cd	-0.424**
Cr	-0.438*
Cu	-0.561*
Pb	-0.240
V	-0.496**
Zn	-0.299

*, *p* <0.05, and **, *p* <0.01.

References

Liu, X., Gu, S., Yang, S., Deng, J., Xu, J., 2021. Heavy metals in soil-vegetable system around E-waste site and the health risk assessment. Sci. Total Environ. 779, 146438.

USEPA (United States Environmental Protection Agency). *Integrated Risk Information System-database*; USEPA: Washington, DC, USA, 2015.
